# Development and effects of tacrolimus-loaded nanoparticles on the inhibition of corneal allograft rejection

**DOI:** 10.1080/10717544.2019.1582728

**Published:** 2019-03-21

**Authors:** Qianni Wu, Dong Liu, Xulin Zhang, Dongni Wang, Meimei DongYe, Wan Chen, Duoru Lin, Fangming Zhu, Weirong Chen, Haotian Lin

**Affiliations:** a State Key Laboratory of Ophthalmology, Zhongshan Ophthalmic Center, Sun Yat-sen University, Guangzhou, China;; b GDHPPCLab, School of Chemistry, Sun Yat–sen University, Guangzhou, China

**Keywords:** Tacrolimus, mPEG-b-PLGA, nanoparticles, keratoplasty, immune rejection

## Abstract

Tacrolimus has been widely applied to prevent organ rejection after transplantation. However, the conventional pharmaceutical formulation of tacrolimus limits its applications in ocular therapy due to its hydrophobicity and low corneal penetrability. We optimized tacrolimus-loaded methoxy poly (ethylene glycol-*block*-poly (d, l)-lactic-*co*-glycolic acid) nanoparticles (TAC-NPs) by simple and effective nanotechnology as a drug delivery system for corneal graft rejection to overcome these drawbacks. The prepared TAC-NPs were 82.9 ± 1.3 nm in size, and the drug loading and encapsulation efficiency were 8.01 ± 0.23% and 80.10 ± 2.33%. Furthermore, New Zealand rabbits were used to analyze the single-dose pharmacokinetics of the TAC-NPs using high-performance liquid chromatography tandem mass spectrometry (HPLC-MS/MS). In rats with allogenic penetrating keratoplasty, the administration of TAC-NPs dispersion drops improved the TAC concentrations in the aqueous humor and cornea, consistent with a significantly higher effective inhibition of IL-2, IL-17, and VEGF expression compared with conventional 0.1% tacrolimus drops. Meanwhile, we also compared two different topical administration methods (including eye drop and subconjunctival injection) of TAC-NPs to maximize the sustained release characteristic of NPs. In summary, the small-sized TAC-NPs enhanced transcorneal permeation and absorption of TAC and more effectively inhibited corneal allograft rejection, which indicated that biodegradable polymeric nanomaterials-based drug delivery system had great potential for improving the clinical therapy efficacy of hydrophobic drugs.

## 1. Introduction

Unfortunately, irreversible rejection constitutes a high proportion (34%) of the reasons for graft failure, especially for penetrating keratoplasty, which is performed for visual improvement (Williams et al., 2008). Notably, tacrolimus has been widely used to prevent organ rejection after transplantation because it has better clinical activity than CsA and is extensively applied to patients who resist corticosteroids or have secondary complications induced by steroids (Kino et al., 1987; Scott et al., 2003). However, challenges still exist in the application of tacrolimus because of its limited aqueous solubility and low corneal penetrability (Tamura et al., 2002). Therefore, transplant recipients require higher drug dosages or frequencies to maintain an effective concentration in the ocular region, which potentiate side effects and economic burden.

Over the past few decades, numerous pharmaceutical technologies, including nanomedicine, have been explored to overcome the deficiencies of tacrolimus (Duncan & Gaspar, 2011; Moghassemi & Hadjizadeh, 2014). Drugs can be absorbed onto the nanoparticle (NP) surface or entrapped inside to enhance their stability, solubility, and cellular penetration. Popular NP systems include liposomes (Honda et al., 2013; Immordino et al., 2003), surfactants (Kagayama et al., 1993), and polymeric NPs (Torchilin 2007; Maeda et al., 2009; Wang et al., 2011), among which polymeric NPs have emerged as one of the most promising drug delivery systems due to their biocompatibility and amphipathy (Barbu et al., 2006; Mandal et al., 2017). Polymeric carriers also have the capacity to maintain therapeutic doses and release drugs in a controlled manner with low toxicity and biodegradability. One of the most attractive polymeric NP for biomedicine applications is poly (lactic-*co*-glycolic acid) (PLGA) (Honda et al., 2013), which does not exhibit electrophysiological or histological toxicity (Moritera et al., 1991; Giordano et al., 1995). Recent comparative studies have indicated that PLGA can nonenzymatically hydrolyze and degrade faster than corresponding homopolymers *in vivo* (Yasukawa et al., 2005) and can thus be formulated as implants, microspheres, and nanocapsules for biomedical applications. In previous studies, Shin et al. (2010) prepared TAC-PLGA NPs with a poly (ethylene glycol) (PEG) surface modification and then administered the prepared NPs to rats intravenously. TAC-PLGA and TAC-PEG-PLGA showed significantly higher targeting efficiencies than conventional tacrolimus.

In summary, this study aimed to develop a nano-sized and biocompatible drug delivery system for tacrolimus, based on an amphiphilic copolymer consisting of methoxy PEG (mPEG) and PLGA. We optimized tacrolimus-loaded mPEG-*b*-PLGA nanoparticles (TAC-NPs) to exhibit higher bioavailability and stability, then topically applied the TAC-NPs to inhibit corneal immunologic rejection after keratoplasty for the first time. The immunosuppressive effect of TAC-NPs dispersion drops was investigated in a rat high-risk allogenic corneal transplantation model, compared with conventional 0.1% TAC drops. Moreover, we also investigated the immune depressive efficacies of two forms of administration involving TAC-NPs eye drops and TAC-NPs subconjunctival injection. This work offers a strategy to prepare small-sized TAC-NPs with biocompatible property, sustained release behavior and better immunosuppressive effects to the treatment of corneal immunologic rejection in rats, which provides promising applications for ocular drug delivery.

## 2. Material and methods

### 2.1. Materials

Poly (ethylene glycol)-*block*-poly (d, l)-lactic-*co*-glycolic acid (mPEG-*b*-PLGA, PEG 2000 Da, PLGA 8000 Da, LA/GA = 75/25) was provided by the laboratory of Professor Fangming Zhu at the School of Chemistry, Sun Yat-sen University, and tacrolimus was obtained from Selleck Chemicals (Shanghai, China). Dialysis bags were purchased from Spectrapor (MWCO 14000, Waltham, MA, USA). Isoflurane inhaled anesthesia (Yipin pharmaceutical Co., LTD, Hebei, China) and 3% sodium pentobarbital solution (Merck, Darmstadt, German) were used to anesthesia animals. Proparacaine hydrochloride eye drops (S.A. ALCON, Fort Worth, TX, USA), Iviz (Bausch Lomb, Shandong, China), and 10-0 black mono nylon sutures (Alcon, Fort Worth, TX, USA) were used in corneal transplantation of rats. Tobramycin eye ointment purchased from Zhongshan Ophthalmic Center (Guangzhou, China). 0.1% tacrolimus eye drops were purchased from Senju Pharmaceutical Co., Ltd. (Osaka, Japan). Rabbit polyclonal anti-CD4/CD8 antibody and horseradish peroxidase (HRP)-conjugated goat anti-rabbit IgG H&L were purchased from Abcam (Cambridge, MA, USA), the Milliplex^®^ xMAP Rat Cytokine/Chemokine kit was purchased from EMD Millipore Corporation (Billerica, MA, USA).

### 2.2. Animals

All research and animal experiments described herein were carried out in accordance with the Association for Research in Vision and Ophthalmology (ARVO) Statement Regarding the Use of Animals in Ophthalmic and Vision Research and abided by the guidelines stipulated by the Animal Experimental Ethics Committee of Zhongshan Ophthalmic Center at Sun Yat-sen University (ethical number: 2017-029). New Zealand albino rabbits (male and female, weighing 2–2.5 kg) were provided by the Huadong Xinhua experimental animal breeding facility (Guangzhou, China) and housed under conventional standard conditions. In addition, Wistar rats (male and female, weighing 200–250) and Sprague-Dawley (SD) rats (male and female, weighing 200–250 g) were specific pathogen-free (SPF) grade and purchased from Jinan Pengyue Experimental Animal Breeding Co., Ltd. (Shandong, China). The rats were housed under SPF standard conditions at the Animal Experimental Center of Zhongshan Ophthalmic Center of Sun Yat-sen University and free fed a standard sterile pellet diet and water.

### 2.3. Preparation and characterization of TAC-NPs

#### 2.3.1. Preparation of TAC-NPs and size measurements

The preparation of TAC-NPs was based on the solvent evaporation method. Briefly, the mPEG-*b*-PLGA copolymers and tacrolimus (mass ratio of 9:1) were dissolved in acetone, and the aqueous solution was added during ultrasonic stirring. After vacuum evaporation and dialysis, the resulting solution was filtered through a 0.22-μm filter to obtain homogeneous NPs at a concentration of 2.5 mg/ml. Blank NPs without tacrolimus were prepared by the same method. The mean particle size and size distribution were recorded by dynamic light scattering (Brookhaven BI-2005M, USA) with a scattering angle of 90 degrees at 25 °C. Each sample was measured in triplicate.

#### 2.3.2. Determination of encapsulation efficiency and drug loading ability

The encapsulation efficiency (EE) and drug loading (DL) were determined by HPLC with a C18 column at 40 °C, with a flow rate of 1 ml/min and a mobile phase comprising a mixture of acetonitrile and 0.05 M phosphoric acid (75: 25, *v*/*v*). EE and DL were calculated using the following equations:

$$\text{EE}\;\text{(\%)}=\frac{\text{mass}\;\text{of}\;\text{FK506}\;\text{in}\;\text{NPs}}{\text{mass}\;\text{of}\;\text{initial}\;\text{FK506}}\times 100\%$$

$$\text{DL}\;\%=\frac{\text{mass}\;\text{of}\;\text{FK506}\;\text{in}\;\text{NPs}}{\text{mass}\;\text{of}\;\text{NPs}}\times 100\%$$

#### 2.3.3. Morphology studies

Transmission electron microscopy (TEM, JEM-1400 Plus, Japan) was used to observe the morphological structure of the TAC-NPs. Briefly, the TAC-NPs were dispensed onto a copper mesh, to which a drop of phosphotungstic acid (1%, *w*/*v*) was applied for staining. After 30 s, the extra dye liquor was absorbed by filter paper and then dried at room temperature.

#### 2.3.4. In vitro release behavior of drug

In vitro release behavior of TAC-NPs was performed using the dialysis method. The TAC-NPs dispersion (1 ml) and 0.1% TAC suspension (1 ml) were respectively placed in dialysis bags. The dialysis bags were both incubated in 25% ethanol/artificial tears (*v*/*v*) to maintain sink conditions at 37 °C and shaken at 90 rpm. At every time intervals, 1 ml of the release medium was removed and replaced by 1 ml of fresh medium. The amount of the TAC was analyzed by HPLC. All the assays were performed in triplicate and results were expressed as a mean value ± SD.

### 2.4. Single-dose pharmacokinetics study

A 25-μl drop of the TAC-NPs dispersion was instilled into the right conjunctival sacs of rabbits. Then, aqueous humor and cornea samples were collected from the right eyes of the rabbits at 0.25, 0.5, 1, 2, 4, 6, 8, 12, and 24 h. A 25-μl TAC-NPs dispersion was subconjunctivally injected into the right eyes of rabbits, and aqueous humor and cornea samples were collected at 0.5, 1, 2, 6, 12, 24, 48, 72, 96, and 120 h. There were three rabbits at each time point, and specimens were immediately placed in liquid nitrogen and stored at −80 °C until HPLC-MS/MS analysis.

### 2.5. High-risk corneal transplantation in rats and postoperative treatment

Only the right eyes of SD and Wistar rats were used in the allogenic grafting experiments (SD to Wistar). Prior to the surgical procedures, all rats were anesthetized by an intraperitoneal injection of 3% sodium pentobarbital solution (1.0–1.5 ml/kg) assistant with isoflurane inhaled anesthesia (1.5–2%) and proparacaine hydrochloride eye drops were used for topical anesthesia. Donor corneas 3.25 mm in diameter were excised with Vannas scissors. The recipient cornea was outlined with 3.0-mm trephine, and then removed. Throughout the entire operation, Iviz was applied to the anterior chamber to maintain stability and protect corneal endothelial cells. Sequentially, the donor buttons were sewn onto the recipient bed with eight interrupted 10-0 sutures and then indwelled in place to aggravate neovascularization and the occurrence of immune rejection. Finally, the anterior chamber was irrigated with sterile physiological saline and restored by sterile small bubbles.

All transplanted rats received tobramycin eye ointment postoperatively were randomly distributed into four groups (12 rats of each group). Group 1 rats, serving as the blank control, received blank mPEG-*b*-PLGA dispersion drops without tacrolimus (25 μl, 2.5 mg/ml) twice a day. Group 2 rats received a subconjunctival injection of TAC-NPs dispersion (25 μl, 2.5 mg/ml, loaded with 0.2 mg/ml TAC) every two days. Group 3 rats were treated with TAC-NPs dispersion drops (25 μl, 2.5 mg/ml, loaded with 0.2 mg/ml TAC) twice a day, and group 4 rats received the conventional 0.1% tacrolimus eye drops (25 μl, 1 mg/ml) twice a day as the positive control. All the treatments described above were administered to the grafted eyes from the 1st to the 28th day after surgery.

### 2.6. Clinical evaluation of corneal transplantation rats

Grafts in each group were observed every other day via a slit-lamp microscope digital system (Topcon, SL-D7/DC-3/IMAGEnet, Japan) with diffuse light and cobalt-blue light examination (after fluorescein staining) until day 28 (six rats of each group). In addition, Holland’s grade (Holland et al., 1994) was used to evaluate corneal graft survival (Herbort et al., 1989), for which the indicators consisted of transparency, edema, and neovascularization. Graft rejection was defined as an opacity score ≥3 or a total score ≥5. Rats with complications, including hemorrhage, cataract, or infection, were eliminated from the study.

### *2.7. HPLC-MS*/*MS assay to determine the tacrolimus concentration in tissues*

In the single-dose pharmacokinetics study, rabbit aqueous humor (30 μl each) and cornea samples were collected as described above. In addition, in corneal transplantation rats, the aqueous humor and corneas of each group were collected on day 14 and day 28 after surgery with three rats at each time point. Aqueous humor (10 μl each) and cornea samples, which had been ground into a powder, were spiked with diazepam (10 ng/ml) as the internal standard and mixed with acetonitrile to precipitate tacrolimus. All the mixtures were vortexed and then centrifuged at 13,000 rpm for 15 min. The upper organic solvent was dried with nitrogen. Then, the residue was redissolved in acetonitrile and injected into the HPLC-MS/MS system for analysis.

### 2.8. Hematoxylin-eosin (HE) and immunohistochemical staining

Rats were randomly executed via narcotic overdose on the 14th and 28th days after the operation. The paraffin-embedded cornea sections were stained with HE. For the immunohistochemistry assay, after incubation with a rabbit polyclonal anti-CD4/CD8 antibody overnight at 4 °C, the sections were incubated with HRP-conjugated goat anti-rabbit IgG H&L and then examined under a microscope (Axioplan2 Imaging, Zeiss).

### 2.9. Luminex^®^ xMAP^®^ technology assay to quantitatively measure immune factors

On the 14th and 28th days after the operation with three rats at each time point, the aqueous humors of each group were aspirated via a 33-gauge needle attached to a microliter syringe (Hamilton, USA) while the rats were anesthetized. Then, the corneas were carefully enucleated, and proteins were extracted using cell lysis buffer (Cell Signaling Technology, USA) supplemented with protease inhibitors (Roche, USA). In this study, Luminex^®^ xMAP^®^ technology (Bio-Rad Laboratories, Inc., USA) and the Milliplex^®^ xMAP Rat Cytokine/Chemokine Magnetic Bead Panel 96-well plate assay (#RECYTMAG-65K) were used to quantify the total protein levels of rat interleukin-2 (IL-2), IL-17, interferon gamma (IFN-γ), and vascular endothelial growth factor (VEGF), and MILLIPLEX^®^ Analyst 5.1 software-1 seat license (MXMSM, Merck, USA) was then used to analyze the data.

### 2.10. Statistical analysis

Results of the Luminex^®^ xMAP^®^ technology and HPLC-MS/MS assays were analyzed by Permutation test using the software R (version 3.5.2, Bell Laboratories, USA), *p*-values less than .05 indicated statistically significant differences. The pharmacokinetic parameters were analyzed using DAS (ver2.1.1).

## 3. Results

### 3.1. Preparation and characterization of TAC-NPs

**Figure 1.**
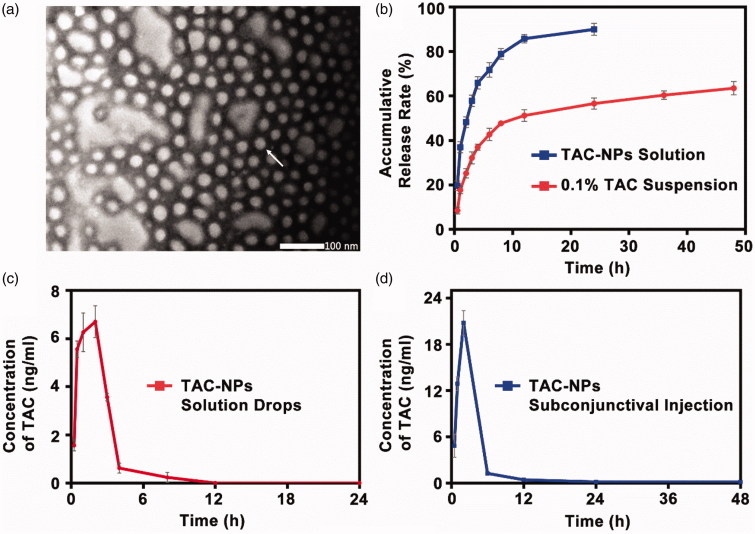
(a) TEM image of TAC-NPs (100,000×, scale bar, 100 nm). (b) In vitro release profiles of TAC-NPs and 0.1% TAC suspension. Pharmacokinetic profile of TAC-NP after a single dose (25 µl, containing 5 µg TAC) topical administrations, including drops (c) and subconjunctival injection (d) in rabbits (data were expressed in mean ± SD, *n* = 3).

TAC-NPs were prepared through the solvent evaporation method assisted with ultrasonication that self-assembled into the core-shell nanostructure in aqueous solution. The hydrophobic drug encapsulated into the lyophobic core and hydrophilic shell enhanced the water solubility and stability. The TAC-NPs were 82.9 ± 1.3 nm in size and had a polydispersity index (PI) of 0.090 ± 0.014 and a zeta potential of −18.90 ± 2.45 mV. The narrow particle size range and high zeta potential value indicated the stability of TAC-NPs in physiological fluids. The particle size also demonstrated enhanced drug permeability, which is the major goal of ocular drug targeting (Sahoo et al., 2008). In addition, morphological analysis of the TAC-NPs, displayed in Figure 1(a), demonstrated TAC-NPs were distributed uniformly without aggregation, and the diameter of particles was in nanometer range.

According to HPLC analysis, the TAC-NP DL and EE values, two important properties of drug-loaded polymeric NPs, were 8.01 ± 0.23% and 80.10 ± 2.33%, which were higher than the typical drug capacity (∼<5%) (Lv et al., 2018). As observed in Figure 1(b), the release profiles for TAC from the TAC-NPs and 0.1% TAC suspension were presented. Compared with the non-encapsulated drug formulations, the drug release behavior of TAC-NPs showed a pronounced sustained property. For 0.1% TAC suspension, more than 85% of TAC was released during 12 h, but only 64% of TAC was released from the TAC-NPs after 48 h. This sustained release behavior might be attributed to the NPs with core/shell structure, where the drug was encapsulated in the hydrophobic core and consistently released via diffusion and the polymeric matrix degradation (Gajendiran et al., 2013; Panyam et al., 2003; Choudhury et al., 2015). On account of its release behavior, TAC-NPs could reduce the administration frequency.

### 3.2. Single-dose pharmacokinetics study

The HPLC-MS/MS method was successfully applied to analyze TAC concentration in corneas and aqueous humor of rabbits after single-dose topical administration of TAC-NPs dispersion (25 μl, containing 5 µg TAC). At 12 h and 24 h, TAC concentrations in cornea tissues were 1.35 ± 0.06 ng/mg and 0.96 ± 0.07 ng/mg after a drop of TAC-NPs, and 2.45 ± 0.17 ng/mg and 0.17 ± 0.04 ng/mg after subconjunctival injection of TAC-NPs. The curves for the mean tacrolimus concentration versus time in the aqueous humor after single-dose topical administration are depicted in Figure 1(c,d). Additionally, concentration-time curves for the TAC-NPs drops administration revealed a maximum concentration of tacrolimus of 6.96 ± 0.59 ng/ml (Figure 1(c)). In addition, concentration-time curves for the TAC-NP subconjunctival injection group revealed a maximum concentration of tacrolimus of 20.80 ± 1.59 ng/ml (Figure 1(d)). Table 1 shows the pharmacokinetic parameters. In the aqueous humor, the AUC_0–24h_, AUC_0–∞_, *T*_1/2_, *T*_max_, and *C*_max_ of TAC-NPs drops were 25.86 ± 1.05 ng h/ml, 26.38 ± 1.14 ng h/ml, 1.20 ± 0.48 h, 1.67 ± 0.58 h, and 6.96 ± 0.59 ng/ml, and those of TAC-NPs subconjunctival injections were 78.61 ± 3.33 ng h/ml, 79.98 ± 1.12 ng h/ml, 11.51 ± 9.94 h, 2.00 h, and 20.80 ± 1.59 ng/ml, respectively.

**Table 1.** The pharmacokinetic parameters of TAC-NPs in the rabbit aqueous humor after single-dose topical administration of TAC-NPs*.

Parameters	TAC-NP subconjunctival injection	TAC-NP dispersion drops
AUC_0–t_ (ng h ml^−1^)	78.61 ± 3.33	25.86 ± 1.05
AUC_0–∞_ (ng h ml^−1^)	79.98 ± 1.12	26.38 ± 1.14
*T*_1/2_ (h)	11.51 ± 9.94	1.20 ± 0.48
*T*_max_ (h)	2.00	1.67 ± 0.58
*C*_max_ (ng mL^−1^)	20.80 ± 1.59	6.96 ± 0.59

* TAC-NPs dispersion (25 μl, 2.5 mg/ml, containing 5 µg TAC).

### 3.3. Clinical evaluation of high-risk corneal transplantation in rats

**Figure 2.**
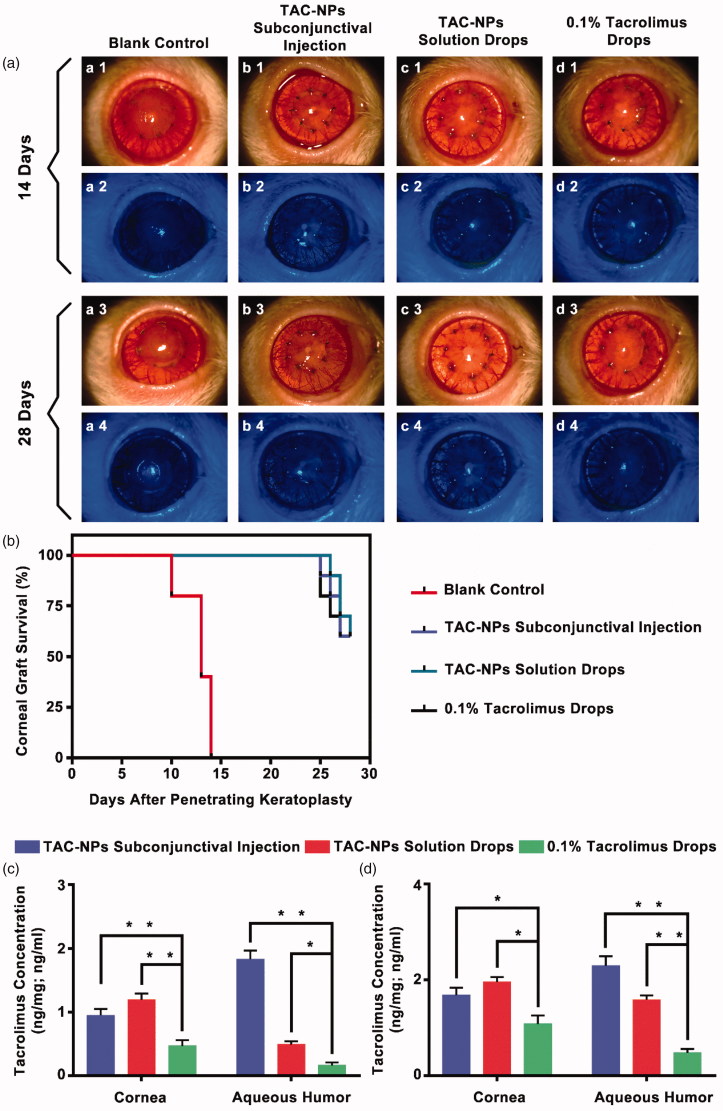
Clinical manifestations and tacrolimus concentration in rats (a) Clinical manifestations (magnification, 250×) of the anterior segment on postoperative day 14 and 28. (b) Survival curves of rat corneal grafts for all groups. The concentrations of tacrolimus in the corneas (ng/mg) and aqueous humor (ng/ml) on postoperative day 14 (c) and 28 (d). Error bars represent the means ± SD. *A statistically significant difference compared to the control groups at the level of *p* < .05 using Permutation test. *N* = 3.

As shown in Figure 2(a), the grafted corneas treated with blank mPEG-*b*-PLGA NPs dispersion drops (blank control) showed pronounced rejection with obvious corneal edema, opacity, thickening, and neovascularization of the grafts. Conversely, the corneas were clear in the other three groups treated with TAC-NPs and 0.1% tacrolimus drops. Additionally, no significant corneal epithelial deficiency was observed upon fluorescein staining in any of the groups as determined by cobalt-blue light examination under a slit-lamp microscope. According to Holland’s grade results, the survival curves are summarized in Figure 2(c). The subconjunctival injection of TAC-NPs dispersion, TAC-NPs drops, and 0.1% tacrolimus drop groups exhibited distinctly prolonged graft survival times compared with those of the blank control group (*p* < .001, *N* = 6).

### *3.4. Tacrolimus concentrations in corneal transplantation rats as determined by HPLC-MS*/*MS*

The tacrolimus concentrations in aqueous humor and cornea samples from all groups were measured by HPLC-MS/MS on postoperative day 14 (Figure 2(c)) and day 28 (Figure 2(d)). In detail, on postoperative day 14, tacrolimus concentrations of TAC-NPs subconjunctival injection group were 0.95 ± 0.10 ng/mg in cornea and 1.83 ± 0.14 ng/ml in aqueous humor; that of TAC-NPs drops group were 1.19 ± 0.10 ng/mg in cornea and 0.49 ± 0.05 ng/ml in aqueous humor; but that of 0.1% tacrolimus drops group were 0.47 ± 0.09 ng/mg in cornea and 0.17 ± 0.05 ng/ml in aqueous humor. On postoperative day 28, tacrolimus concentrations of TAC-NPs subconjunctival injection group were 1.68 ± 0.16 ng/mg in cornea and 2.29 ± 0.20 ng/ml in aqueous humor; that of TAC-NPs drops group were 1.95 ± 0.11 ng/mg in cornea and 1.58 ± 0.09 ng/ml in aqueous humor; however that of 0.1% tacrolimus drops group were 1.08 ± 0.18 ng/mg in cornea and 0.48 ± 0.08 ng/ml in aqueous humor.

Because the drug loading (DL) of TAC-NPs was 8.01 ± 0.23%, 2.5 mg/ml TAC-NPs loaded nearly 0.2 mg/ml tacrolimus, which was lower than conventional 0.1% tacrolimus drops (1 mg/ml). However, the tacrolimus concentration in the cornea and aqueous humor of TAC-NPs treated groups were significantly higher than in the 0.1% tacrolimus drops group (*p* < .05). Therefore, mPEG-b-PLGA NPs noticeably improve the penetrability and bioavailability of tacrolimus in ocular.

### 3.5. Histopathological analysis

**Figure 3.**
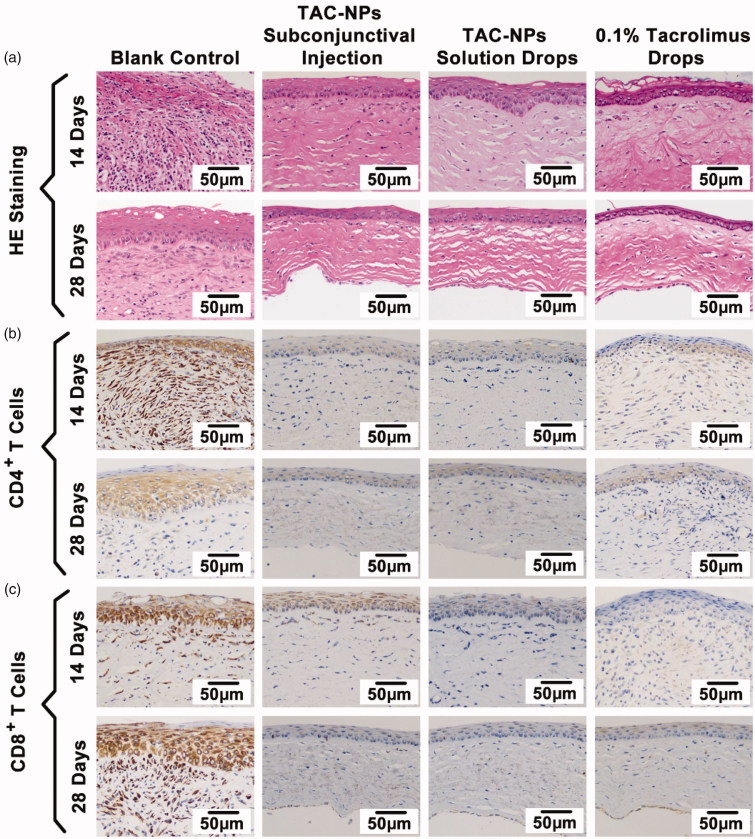
HE and immunohistochemical staining of corneal grafts (magnification, 200×). (a) HE staining, (b) immunohistochemical staining of CD4**^+^** T cells, and (c) immunohistochemical staining of CD8^+^ T cells. Greater numbers of CD4**^+^** and CD8^+^ T cells (stained in brown) were observed in the blank control group than in the TAC-NP-treated and 0.1% tacrolimus-treated groups.

The histopathological analysis of corneas, including HE and immunohistochemical staining, revealed signs of immune-mediated graft rejection in all groups (Figure 3). As shown by the HE staining, the allografts in the blank control group exhibited substantial inflammatory cell accumulation, thickening, stroma fibrosis, and edema on postoperative day 14 and 28. By contrast, the drug-treated groups exhibited fewer inflammatory cells as well as less fibrosis, thickening, and edema. In addition, immunohistochemical staining of the corneal grafts showed reduced numbers of CD4^+^ T cells and CD8^+^ T cells, which are closely related to T cell immune responses, in the groups receiving the subconjunctival injection of the TAC-NPs dispersion, TAC-NPs drops, and 0.1% tacrolimus drops on days 14 and 28.

### 3.6. Quantitative measurement of immune factors using Luminex^®^ xMAP^®^ technology

**Figure 4.**
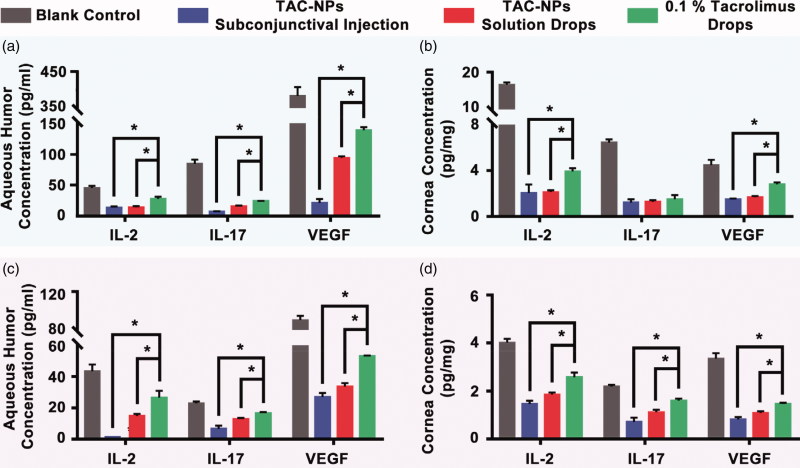
Concentrations of IL-2, IL-17, and VEGF in aqueous humor and cornea on postoperative days 14 (a, b) and 28 (c, d). TAC-NPs inhibited immune reactions more effectively than conventional 0.1% tacrolimus (*p* < .05). Error bars represent the means ± SD. *A statistically significant difference compared to the control groups at the level of *p* < .05 using Permutation test. *N* = 3.

The levels of IL-2, IL-17 and VEGF were detected in the aqueous humors and corneas of all groups on postoperative day 14 (Figure 4(a,b)) and 28 (Figure 4(c,d)) with three rats at each time point. In the cornea and aqueous humor, the expression levels of IL-2, IL-17, and VEGF in the blank control group were significantly higher than those in the TAC-NPs-treated and 0.1% tacrolimus drops groups on postoperative days 14 and 28. Furthermore, both TAC-NPs subconjunctival injection group and TAC-NPs drops group inhibited IL-2, IL-17 and angiogenesis to a greater extent than the 0.1% tacrolimus drops (*p* < .05). Additionally, IFN-γ expression was only detected in the corneas of the blank control group (18.64 ± 0.85 ng/mg) on postoperative day 14 (below the limit of detection in the other groups).

## 4. Discussions

Conventional immunosuppressive drugs (particularly tacrolimus) have low bioavailability when applied topically for ophthalmology purposes, not only because of their limited corneal penetration ability but also because of elimination by tear irrigation and drainage of the nasolacrimal duct. Accordingly, a new topical ophthalmic agent, which could overcome the deficiencies of tacrolimus, is particularly important for ocular drug delivery systems. The use of nanomedicine for diagnosis and therapy has rapidly advanced in recent years. Polymeric NPs greatly improve the tissue penetrability of hydrophobic drugs, due to their mucoadhesive nature and retention abilities (Qiu & Bae 2006; Honda et al., 2013), which enhance the contact between drugs and ocular surfaces, and amphiphilicity that generates stability of hydrophobic drugs in aqueous solutions. Comparative studies showed that PEG and PLGA are commonly applied in biomedicine with low toxicity, Souza and Kalam both suggested that TAC-PLGA could prolong drug release with no evidence of toxic effects in the rabbits’ eye (Souza et al., 2014; Kalam and Alshamsan, 2017). Chen et al. (2017) proved that PLGA-PEG-PLGA loaded with US597 enhanced its effects and bioavailability in cancer therapy. Therefore, for the first time, we present the application of biocompatible TAC-NPs in high-risk corneal transplantation rats and demonstrate TAC-NPs positively improve the immune inhibition effects of TAC.

Notably, we investigated the immunosuppressive effect of TAC-NPs dispersion drops in a rat high-risk allogenic corneal transplantation model, compared with conventional 0.1% TAC drops, which was commonly used in clinical treatment of corneal rejection. According to our postoperative clinical observations in keratoplasty rats, diffused light and cobalt-blue light analysis showed no signs of conjunctival congestion, edema, cornea epithelial deficiency, or other ocular toxic reactions in the TAC-NPs-treated groups, which was due to the biodegradability of the mPEG-*b*-PLGA copolymer. Furthermore, the degradation products lactic acid and glycolic acid of mPEG-*b*-PLGA were able to be metabolized by the body (Hideya and Yuichiro, 2001). In addition, immunological rejection was generally detectable at 10–14 days after surgery in the blank control group, whereas the TAC-NPs-treated groups exhibited better transparency, slight edema and neovascularization reductions of the cornea until the end of the observation period.

Based on the TAC concentrations detected by HPLC-MS/MS in cornea and aqueous humor samples from the corneal transplantation rats, TAC-NPs drops (0.2 mg/ml TAC) group exhibited significantly higher (*p* < .05) drug concentrations in ocular tissues than the conventional 0.1% tacrolimus drops group. Therefore, mPEG-*b*-PLGA NPs greatly improved the trans-corneal permeation and absorption of TAC. These significantly high TAC concentrations in ocular related to the smaller size and stability of the mPEG-*b*-PLGA NPs, which could prolong the TAC retention on the corneal surface and further enhanced drug trans-corneal uptake. Furthermore, upon reaching the intraocular tissues, the TAC-NPs sustained tacrolimus release via drug diffusion and polymer degradation. However, the conventional TAC drops are commonly in suspension form and easily drained from the ocular surface with poorly ability to penetrate the cornea (Zeng et al., 2016).

Tacrolimus inhibits allograft rejection primarily by activating T cells after binding to the cellular receptors (FKBP12), thus blocking the activity of calcineurin and regulating the transcription of lymphokine IL-2. Therefore, IL-2 is a vital target for the activation of a TCR-mediated signaling transmission pathway (Scott et al., 2003). Besides, inflammatory cytokine (such as IFN-γ and IL-17) also possess immunoregulatory. VEGF participates in the neovascularization process and promotes the contact between donor antigens and recipient blood lymphocytes, which enhances cornea immunological rejection (Williams et al., 2008; Qazi et al., 2010; Di Tommaso et al., 2012). Therefore, to analyze the immunosuppressive effects of TAC-NPs, we performed biochemical experiments to measure the expression levels of IL-2, IFN-γ, IL-17, and VEGF in anterior segment of rats using Luminex^®^ xMAP^®^ technology. Here, the levels of immune factors (including IL-2, IL-17, and VEGF) in TAC-NPs drops group were all significantly lower than 0.1% tacrolimus drops group (*p* < .05). Additionally, IFN-γ is one of the acute immune rejection indicators, produced by reactive regulatory T cells. About 14 days later, in the blank control group, the degree of inflammatory cells infiltration and cornea edema were reduced instead of a large number of fibroblasts and graft scarring (Tanaka et al., 2005). Besides, TAC-NPs and 0.1% tacrolimus drops persistently inhibited allograft rejection. Therefore, IFN-γ expression was only detected in the corneas of the blank control group on postoperative day 14. Consequently, topical TAC-NPs treatments displayed better and more stable curative effects and resulted in higher corneal drug concentrations than conventional 0.1% tacrolimus drops in a high-risk corneal allograft transplantation rat model.

Furthermore, we also compared two different topical administration methods (including eye drop and subconjunctival injection) to maximize the sustained release characteristic of TAC-NPs dispersion, because subconjunctival injection could prolong corneal allograft survival than eye drops (Mills et al., 1995; Hikita et al., 1997; Dickey et al., 1993; Okada et al., 1996; Fei et al., 2008). The efficacy of TAC-NPs subconjunctival injection (25 µl, every other day) was proven to be stable and better than TAC-NPs eye drop (25 µl, twice a day), possibly due to the route of ocular administration. First, the subconjunctival space may contain larger aqueous solution volumes than conjunctival sac. Additionally, the drug can follow the anterior pathway, transscleral pathway, and systemic circulation via the choroid, thus reaching ocular tissues at therapeutic concentrations (Mandal et al., 2017). Above all, based on the advantages mentioned above, subconjunctival administration facilitated the retention of TAC-NPs and sustained release of TAC.

## 5. Conclusions

The present study optimized TAC-NPs, which were nano-sized and biocompatible. We further confirmed the drug release behavior of TAC-NPs showed a pronounced sustained property in vitro, and we also analyzed the pharmacokinetic parameters of TAC-NPs after single-dose topical administration in rabbits. Furthermore, in rats with allogenic penetrating keratoplasty, TAC-NPs sustained released TAC at the targets and delivered higher drug concentrations into the cornea and aqueous humor than the conventional 0.1% tacrolimus drops. Moreover, TAC-NPs efficiently inhibited corneal allograft rejection in rats, as they reduced the IL-2, IL-17, and VEGF expression levels in the cornea and aqueous humor, which was more effective than 0.1% tacrolimus drops. Therefore, due to their better biocompatibility and sustained drug release, mPEG-*b*-PLGA NPs have great potential for improving the clinical therapy efficacy of hydrophobic drugs for ocular diseases.
